# Considering ABO Incompatible Living Donor Kidney Transplantation Before Deceased Donor Kidney Transplantation in Children: A Letter to the Editor

**DOI:** 10.3389/ti.2023.11613

**Published:** 2023-09-18

**Authors:** Alicia Paessler, Jelena Stojanovic

**Affiliations:** ^1^ Great Ormond Street Hospital for Children NHS Foundation Trust, London, United Kingdom; ^2^ Institute for Child Health, University College London, London, United Kingdom

**Keywords:** ABO incompatible, living donation, paediatric, kidney transplantation, outcomes

Dear Editors,

Advances in neonatal and metabolic medicines have brought up a new profile of paediatric kidney transplant recipients. More children born with congenital anomalies of the kidneys and urinary tract and metabolic conditions are surviving outside of infancy. Some of those children are needing renal replacement therapy since early childhood and therefore are considered as transplant candidates with a high likelihood of needing more than one kidney transplant in their life. Recent meta-analysis states that pre-emptive transplantation has advantages in overall patient and allograft outcomes over transplantation done following a period on dialysis [1].

In the UK there are currently 101 children waiting for a kidney transplant from a deceased donor [2]; this number is even higher in the EuroTransplant region at 138 children [3]. However, the number of children receiving a deceased donor kidney each year is far lower with only 52 children receiving a deceased donor kidney in the UK in 2022 (ranging from 42–60 deceased donor transplants per year over the last decade) [2]. The average waiting time in the UK in the last year for children has been 270 days although this has ranged from 258 to 342 days in the last decade [2]. We also know that allografts coming from living donors have better outcomes than deceased donor allografts and so is the preferred transplant option [4, 5]. However, not all children have suitable living donors that are blood group compatible. In such cases, most children go on to the deceased donor waiting list and can wait a long time before a kidney becomes available, which may still lead to poorer outcomes compared to if they had a living donor [2].

Given the scarcity of deceased donor organs and increasing waiting times, there has been extensive research into ABO incompatible (ABOi) transplantation from a living donor since the 1980s, as a way of increasing donor pool and as a possible alternative to deceased donation. While the studies initially focused on adult donors and recipients, over recent years more evidence has been produced to support this practice for paediatric recipients. An analysis of the Japanese Kidney Transplant registry was published in 2018 which described the results of 102 children who had undergone ABOi kidney transplants from living donors. The outcomes of these recipients were compared to the outcomes of the children on their registry who had undergone ABO compatible living donor transplants, with no difference found in patient or allograft survival between the two groups [6]. Initially the pre-transplant protocol for ABOi transplants included recipient splenectomies prior to or at the time of transplantation [7]. This approach has further developed since then and excellent results have been achieved using protocols with medicines, such as Rituximab for so called “medical splenectomies.” The largest paediatric transplant center in the UK shared their experience of a tailored desensitisation protocol where immunosuppression is based on the recipients’ level of ABO antibody titres pre-transplant. It involves the use of Rituximab ± immunoadsorption and/or double filtration plasmapheresis [8] if titers are 1:8 or higher. Several centers in the UK reported on outcomes of ABOi kidney transplantation with a cohort of 23 children and similarly found no difference in patient or allograft survival, acute rejection or graft function compared to ABO compatible living donor transplants [8, 9]. Other centers in Sweden [10] and Japan [11] using a similar approach to desensitisation have also shared equally encouraging results. Some studies have even found that infants with low antibody titres prior to ABOi transplantation did not require any pre-transplant desensitisation to achieve excellent results [10]. In all studies where Rituximab was used, it was shown that the use of Rituximab pre-transplant was not associated with an increased risk of infection or any other complications either, confirming safety of this drug to be used in ABOi kidney transplant programmes.

Given the increasing evidence of positive outcomes following ABOi kidney transplantation in children, and evidence of better allograft survival of kidneys coming from living donors, perhaps it is time to consider ABOi living donor kidney transplantation in children before being listed for deceased donor organs. To date, there is no prospective study comparing the outcomes between those receiving ABOi living donor transplants and those receiving ABO compatible transplants from a deceased donor. However, the evidence that we do have in the literature so far, supports the idea that ABOi transplants should be considered in the paediatric population as a transplant option prior to proceeding with a transplant from a deceased donor, as it has the potential to lead to better patient and allograft outcomes.

## Data Availability

Publicly available datasets were analyzed in this study. This data can be found here: https://statistics.eurotransplant.org/; https://www.odt.nhs.uk/statistics-and-reports/annual-activity-report/.
